# Integrated Transcriptomic Analyses and Experimental Validation Identify TSPO as a Mitochondria‐Associated Therapeutic Target in Myocarditis

**DOI:** 10.1155/mi/2395578

**Published:** 2026-09-02

**Authors:** Yaxue Xie, Yingnan You, Yanjie Jiang, Xiuyun Duan, Bo Han

**Affiliations:** ^1^ Department of Pediatrics, Shandong Province Hospital, Shandong University, Jinan, Shandong, 250021, China, sdu.edu.cn; ^2^ Department of Pediatrics, Shandong Province Hospital Affiliated to Shandong First Medical University, Jinan, Shandong, 250021, China, sph.com.cn; ^3^ Department of Pediatrics, Clinical Research Center for Children’s Health and Disease Office, Shandong Provincial, Shandong Provincial Hospital, Jinan, Shandong, 250021, China, sph.com.cn

**Keywords:** bioinformatic analysis, macrophages, mitochondrial metabolism, myocarditis, TSPO

## Abstract

Myocarditis is characterized by inflammatory cell infiltration and myocardial injury, yet current treatments remain largely empirical and nonspecific. Given the heart’s high energy demand and the essential role of mitochondria in cellular metabolism, investigating mitochondria‐related genes may provide novel therapeutic insights. In this study, we integrated bulk RNA sequencing data from CVB3 viral myocarditis (VMC) model with mitochondrial gene sets from MitoCarta3.0 and human myocarditis‐related genes from GeneCards. Through comprehensive bioinformatics analyses, including weighted gene coexpression network analysis (WGCNA), random forest (RF), and SHapley Additive exPlanations (SHAP), we identified mitochondrial metabolism‐related genes associated with myocarditis. Single‐cell RNA sequencing (scRNA‐seq) analysis further revealed that macrophages were the predominant infiltrating immune population, with Tspo expressed in myocarditis macrophages. Tspo knockdown enhanced inflammatory responses and disrupted mitochondrial structure and function, whereas treatment with the Tspo ligand Ro5‐4864 exerted the opposite effects. Consistently, administration of Ro5‐4864 in a VMC mouse model mitigated inflammation and preserved cardiac function. Collectively, our findings identify Tspo as a novel mitochondria‐associated protective response factor in myocarditis, providing new insights into its pathophysiological mechanisms and highlighting Tspo as a promising therapeutic target for future intervention.

## 1. Introduction

Myocarditis, which is characterized by inflammation of the myocardium, is a major contributor to the development of dilated cardiomyopathy and heart failure [1, 2]. Viral infections represent the most common etiological agents, with enteroviruses‐particularly coxsackievirus B3 (CVB3)‐being the most extensively studied [1, 3]. Despite substantial progress in understanding myocarditis pathogenesis, current therapeutic strategies remain largely symptomatic and etiological, with no specific targeted treatments available. This limitation is primarily due to the complexity of immune cell infiltration and the multifactorial nature of myocardial inflammation [4, 5]. Therefore, identifying novel molecular targets is essential to improve the treatment and management of myocarditis.

Mitochondria are ubiquitous double‐membrane organelles in eukaryotic cells, consisting of the outer mitochondrial membrane and inner mitochondrial membrane. They serve as the cellular “powerhouses,” generating energy through oxidative phosphorylation (OXPHOS), a process that is particularly vital for meeting the high metabolic demands of cardiac tissue [6]. Beyond their canonical role in energy metabolism, mitochondria act as central hubs for cellular signaling, especially in the regulation of inflammation and immune responses [7]. Reactive oxygen species (ROS), produced as by‐products of OXPHOS, function as critical modulators of mitochondrial antiviral signaling pathways during viral infections [6]. Moreover, disruption of mitochondrial homeostasis‐such as a reduction in mitochondrial membrane potential‐can trigger the release of cytochrome c, leading to apoptosis [8, 9]. Given these diverse roles, mitochondrial‐associated genes (MtGs) are likely to play crucial roles in the pathogenesis of myocarditis. Indeed, in viral myocarditis (VMC) models, substantial mitochondrial abnormalities have been observed, including mtDNA deletions, loss of mitochondrial membrane phospholipids [10], decreased respiratory chain complex activities, and excessive ROS production [11]. Therefore, identifying dysregulated MtGs through bioinformatic approaches provides a promising avenue for uncovering novel therapeutic targets in myocarditis.

In this study, we aimed to elucidate the relationship between myocarditis and MtGs using an integrative bioinformatics approach. This included data from the GEO, MitoCarta3.0, and GeneCards databases, the construction of a weighted gene coexpression network analysis (WGCNA), and the application of machine learning techniques. This analysis suggests that Tspo is a mitochondria‐associated protective response factor potentially involved in the pathogenesis and progression of myocarditis. Furthermore, single‐cell RNA sequencing (scRNA‐seq) analysis revealed that macrophages exhibited the most prominent transcriptional alterations in myocarditis, with Tspo relatively enriched in macrophages. To further validate its functional relevance, we performed in vitro experiments using RAW264.7 macrophages and in vivo studies in a CVB3‐induced myocarditis mouse model. These experiments demonstrated that Tspo plays a protective role in regulating inflammation and mitochondrial function during myocarditis progression. This study enriches the theoretical understanding of mitochondrial genes in myocarditis pathogenesis, provides a comprehensive experimental basis for elucidating the mechanisms of their expression dysregulation, and offers new insights and potential targets for future targeted therapies.

## 2. Results

### 2.1. Dysregulation of Mitochondria‐Related Biological Processes and Screening of Myocarditis‐Associated Mitochondrial Genes

Previous studies have highlighted the critical role of mitochondrial metabolism‐related genes in the pathogenesis of myocarditis [12]. To further elucidate the association between MtGs and myocarditis, we established a series of bioinformatics analysis pipelines to identify mitochondria‐related genes potentially involved in the disease and conducted both in vivo and in vitro validations.

**Figure 1:**
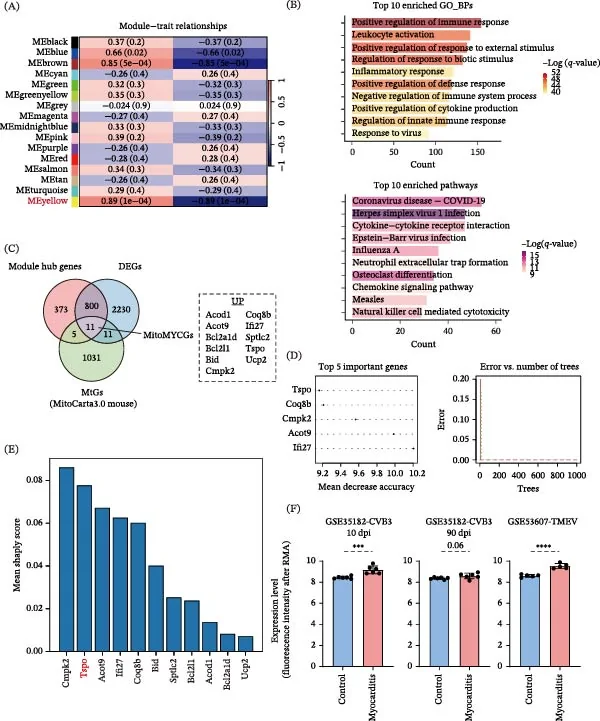
Screening and validation of mitochondrial genes contribute to myocarditis. (A) Relationships between modules and disease status, and the corresponding *p* values in GSE248521. Yellow module (MEYellow) displays the highest correlation with myocarditis (red). (B) Top 10 enriched GO BPs and KEGG pathways of hub module. (C) Overlapping 1189 hub module genes and 3052 DEGs obtained from GSE248521, together with 1140 MtGs (from MitoCarta3.0), identified 11 upregulated MitoMYCGs. (D) Identification of top 5 genes through random forest analysis. (E) SHAP feature importance presented based on the mean absolute SHAP value of each gene. (F) Bar plots of Tspo between myocarditis and control in the 10 dpi and 90 dpi CVB3 induced myocarditis‐GSE35182 and TMEV‐induced myocarditis‐GSE53607.  ^∗∗∗^ *p* value < 0.001;  ^∗∗∗∗^ *p* value < 0.0001; BPs, biological processes; DEGs, differentially expressed genes; MitoMYCGs, mitochondrial genes contribute to myocarditis; MtGs, mitochondrial metabolism‐related genes; ns, nonsignificant; SHAP, SHapley Additive exPlanations.

WGCNA was applied to identify gene modules correlated with disease status. The soft‐thresholding power was optimized to 16, at which the scale‐free topology fit index (*R* ^2^) reached 0.85, ensuring an appropriate network topology (Figure S1). A total of 16 distinct gene modules were identified (including the gray module containing unassigned genes). Among these, the yellow module (MEyellow) exhibited the strongest positive correlation with myocarditis (*R* > 0, *p* < 0.05; Figure 1A) and was therefore selected for downstream analyses.

Hub genes within the MEyellow module were defined using stringent criteria: gene significance (|GS| ≥ 0.50) and module membership (|MM| ≥ 0.60). A total of 1189 hub genes satisfying these thresholds were designated as myocarditis‐associated genes and subjected to further analyses (Table S4). Functional enrichment analysis of MEyellow hub genes revealed significant enrichment in biological processes primarily related to immune and inflammatory regulation‐key mechanisms underlying myocarditis pathogenesis [13, 14] (Figure 1B). Consistently, Kyoto Encyclopedia of Genes and Genomes (KEGG) pathway analysis showed that the top enriched pathways were predominantly associated with viral infection and immune‐inflammatory signaling (Figure 1B), both of which are well‐established contributors to myocarditis, particularly in viral subtypes [1, 13].

To identify MitoMYCGs, we intersected 811 differentially expressed hub genes (*p* < 0.05, |log_2_ (fold change, FC) | ≥ 0.5) with 1140 MtGs retrieved from the MitoCarta3.0 database. This intersection yielded 11 overlapping MitoMYCGs (Figure 1C), including Acod1, Acot9, Bcl2a1d, Bcl2l1, Bid, Cmpk2, Coq8b, Ifi27, Sptlc2, Tspo, and Ucp2.

### 2.2. Screening, Validation of Hub Genes, and SHAP Value Investigation

To identify the most impactful MitoMYCGs, the 11 previously identified MitoMYCGs were subjected to random forest (RF) analysis. This step prioritized the top 5 genes with the highest relevance: Acot9, Cmpk2, Coq8b, Ifi27, and Tspo (Figure 1D). While the feature importance scores from RF provided preliminary insights into the genes’ relative influence, the inherent limitation of RF in interpretability prompted further analysis. To address this issue and quantitatively characterize the contribution of each hub MitoMYCG to myocarditis‐related prediction, we further conducted SHapley Additive exPlanations (SHAP) analysis based on the RF model built using the 11 candidate genes. As shown in Figure 1E, SHAP‐based feature importance ranked genes according to their mean absolute SHAP values, which reflect their overall contribution to the prediction model. Among the 11 candidate genes, Cmpk2 (0.085868), Tspo (0.0775), Acot9 (0.067014), Ifi27 (0.062361), and Coq8b (0.06) displayed relatively higher SHAP values. Notably, these genes were consistent with the top five genes identified by RF analysis, further indicating that they may have a certain degree of importance to myocarditis. Given that the MitoMYCGs were derived from mouse datasets, we next sought to identify conserved MitoMYCGs with relevance to human myocarditis. Human myocarditis‐related genes were retrieved from the GeneCards database, and an intersection with the top 5 mouse MitoMYCGs revealed that only Tspo overlapped, suggesting that Tspo may have a certain degree of cross‐species relevance. For validation, we analyzed its expression in two independent myocarditis datasets, CVB3‐induced myocarditis set GSE35182 (including 10 and 90 dpi) and Theiler’s murine encephalomyelitis virus (TMEV)‐induced myocarditis set GSE53607. As shown in Figure 1F, Tspo expression tended to be elevated in the myocarditis group (*p* < 0.05 at 10 dpi in GSE35182 and GSE53607; *p* = 0.06 at 90 dpi in GSE35182). Considering that different viral models, disease stages, and data preprocessing methods may significantly affect transcriptomic characteristics, these external datasets should be interpreted primarily as providing complementary support across different conditions rather than as direct replications under fully homogeneous settings.

Based on the combined results of RF and SHAP, a certain degree of cross‐species relevance, and validation in external datasets, Tspo was selected as the key MitoMYCG for subsequent investigation of its role in myocarditis pathogenesis.

### 2.3. Cell Variation Analysis of the scRNA‐Seq Dataset and In Vivo Validation

**Figure 2:**
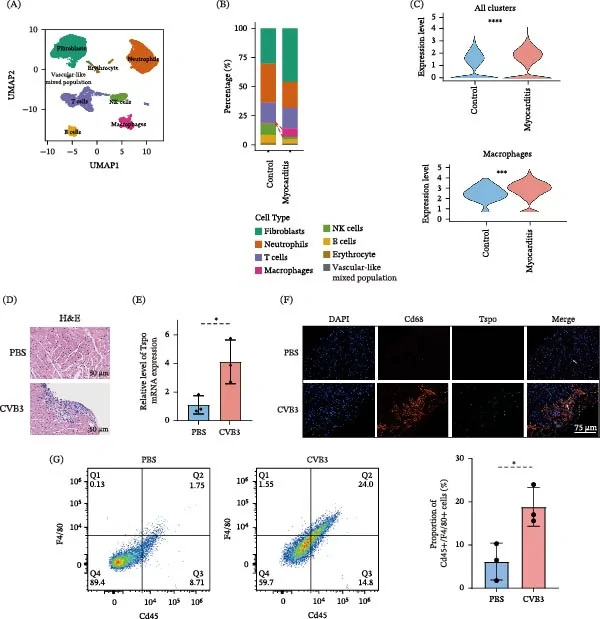
Comprehensive analysis of scRNA‐seq data. (A) Cell type annotation of clusters. 8 dominating cell types were distinguished according to canonical cell markers. (B) The proportion of 8 cell types in myocarditis and control, in which macrophages are the immune cells that have the highest variation. (C) Violin plots of Tspo in all clusters and macrophages between myocarditis and control. (D) H&E staining of heart tissues from mice with and without CVB3 infection (scale bar = 50 μm). (E) Relative mRNA expression of Tspo in heart tissues of mice with and without CVB3 infection (*n* = 3). (F) Representative immunofluorescence images depicting the colocalization between Tspo (green) and the classical marker of macrophage‐Cd68 (red; scale bar = 75 μm). White arrows indicate macrophage in the PBS group and the colocalization of Cd68 and Tspo in the CVB3 group. (G) Flow cytometry was used to detect Cd45 and F4/80 double‐positive cells in mouse cardiac tissue. The proportion of Cd45+F4/80+ cells was significantly increased in the CVB3 group compared with the control group.  ^∗^ *p* value < 0.05;  ^∗∗∗^ *p* value < 0.001;  ^∗∗∗∗^ *p* value < 0.0001; H&E, hematoxylin and eosin; scRNA‐seq, single‐cell RNA sequencing.

Myocarditis is marked by infiltration of immune cells. To ascertain which immune cell types contribute the most to the development of myocarditis, scRNA‐seq data containing two myocarditis and two control samples were downloaded from GSE174458. After performing quality control (Figure S2), a total of 16,235 single cells were preserved for further analysis, with 5705 cells from control and 10,530 cells from myocarditis. Based on classic cell markers (Figure S3), 8 major cell types, namely B cells, erythrocytes, fibroblasts, macrophages, neutrophils, NK cells, T cells, and vascular‐like mixed population, were annotated; however, no clear separation was found for cardiomyocytes (Figure 2A). The composition ratio of each cell type was quantified (Figure 2B and Table S3). Notably, macrophages are the most infiltrating immune cells in myocarditis, which function in the pathogenesis of myocarditis [15, 16]. To evaluate the hub MitoMYCGs expression in macrophages, we utilized the “FindMarkers” function in the Seurat R package to evaluate the relative gene expression levels (Figure 2C and Table 1). According to the results, Tspo showed significant change in macrophages between myocarditis and control group (Figure 2C and Table 1; *p* value = 2.14E‐04, avg.log_2_FC = 0.631). Besides, Figure S4 illustrates both the distribution pattern and expression levels of Tspo across all identified cell clusters per sample. This analysis showed that macrophages consistently exhibited a relatively high Tspo expression across samples. However, because only two biological replicates were available per group, the sample‐level comparison between control and myocarditis samples was descriptive and did not support a robust inference regarding between‐group differences.

**Table 1:** Differential analysis of Tspo in GSE174458 between myocarditis and control in all clusters and macrophages.

Clusters (myocarditis vs control)	Gene	*p*‐value	avg.log2FC	pct.1	pct.2
All clusters	Tspo	9.80E‐126	0.687	0.71	0.596
Macrophages	Tspo	2.14E‐04	0.631	0.891	0.931

Abbreviations: avg.log2FC, log fold‐change of the average expression between the two groups; pct.1, the percentage of cells where the gene is detected in the myocarditis group; pct.2, the percentage of cells where the gene is detected in the control group.

To further corroborate this finding, CVB3 was used to induce myocarditis in mice. Haemtoxylin and eosin (H&E) staining showed robust immune cell accumulation in the hearts of CVB3‐injected mice (Figure 2D). The reverse transcription quantitative polymerase chain reaction (RT‐qPCR) results indicate an upregulation of Tspo in the hearts of CVB3‐injected mice (Figure 2E). In addition, we validated the expression of the other top‐ranked genes in the heart tissue of CVB3‐induced myocarditis mice, and the results showed that these genes were significantly upregulated in the CVB3‐infected group (Figure S5). At the same time, fluorescence images of heart sections demonstrate an upregulation of Tspo preferentially expressed in the macrophages (indicated by Cd68) of the CVB3 group (Figure 2F), and the degree of its colocalization with CD68 varies across distinct lesion regions. Consistently, flow cytometric analysis of mouse cardiac tissue showed that the proportion of Cd45^+^F4/80^+^ cells was significantly higher in the CVB3 group than in the control group (Figure 2G).

In summary, our results indicated macrophages increased significantly in myocarditis, and Tspo was expressed in myocarditis macrophages. We speculate that Tspo plays an important role in the inflammatory response of macrophages.

### 2.4. Tspo Plays an Anti‐Inflammatory Role in Macrophages

**Figure 3:**
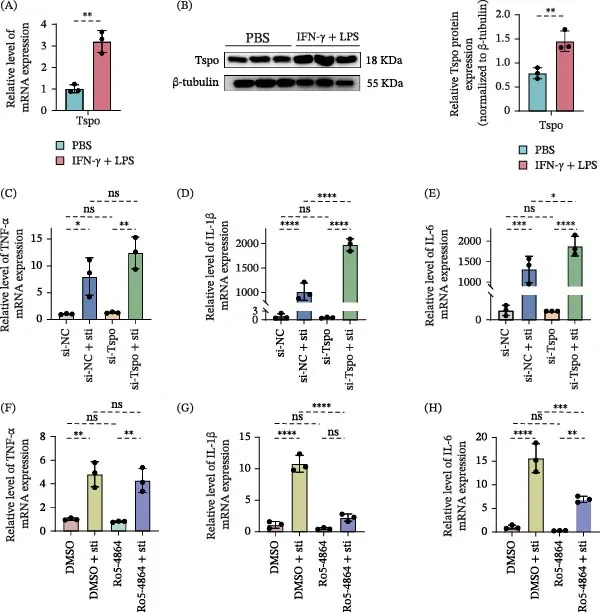
Relationship between Tspo and inflammation. (A) RT‐qPCR of Tspo in RAW264.7 cells between the IFN‐γ+LPS and PBS groups. (B) Western blot was performed to examine Tspo protein levels in the PBS group and IFN‐γ+LPS group. Densitometric quantification was conducted after normalization to β‐tubulin. (C–E) Macrophages with Tspo knockdown displayed enhanced inflammatory responses upon stimulation relative to si‐NC control (*n* = 3). (F–H) Treatment with the Tspo agonist Ro5‐4864 alleviates inflammatory responses in stimulated RAW264.7 macrophages (*n* = 3).  ^∗^ *p* value < 0.05,  ^∗∗^ *p* value < 0.01;  ^∗∗∗^ *p* value < 0.001;  ^∗∗∗∗^ *p* value < 0.0001; DMSO + sti, treatment with DMSO and subsequently stimulated with IFN‐γ and LPS; ns, nonsignificant; Ro5‐4864+sti, treatment with Ro5‐4864 and subsequently stimulated with IFN‐γ and LPS; si‐NC, siRNA negative control; si‐NC+sti, transfected with siRNA negative control and subsequently stimulated with IFN‐γ and LPS; si‐Tspo, siRNA targeting Tspo; si‐Tspo+sti, transfected with siRNA‐Tspo and subsequently stimulated with IFN‐γ and LPS.

To elucidate the function of Tspo in macrophage‐mediated inflammation, an in vitro model was established by stimulating RAW264.7 cells with 1 μg/mL lipopolysaccharide (LPS) and 20 ng/mL interferon‐γ (IFN‐γ) (Figure S7). The expression levels of Tspo in these macrophages were subsequently assessed using RT‐qPCR and Western blot. As shown in Figure 3A,B, Tspo expression was significantly upregulated in the LPS + IFN‐γ group compared to the PBS group at both mRNA and protein levels (*p* < 0.05).

To further explore the biological function of Tspo in macrophages during inflammation, we transfected RAW264.7 cells with siRNA targeting Tspo (si‐Tspo) or a negative control (si‐NC). The transfection efficiency is shown in Figure S8 at both mRNA and protein levels. Knockdown of Tspo resulted in a significant increase in the levels of inflammatory cytokines, IL‐1β and IL‐6 (Figure 3D,E), an effect that could be reversed by treatment with the Tspo agonist Ro5‐4864 (Figure 3G,H). Although the difference did not reach statistical significance, Tspo knockdown tended to elevate tumor necrosis factor‐α (TNF‐α) expression (Figure 3C), whereas pretreatment with Ro5‐4864 reversed this elevation (Figure 3F). Collectively, these findings suggest that Tspo may play a protective role in macrophages by modulating inflammatory cytokine production.

### 2.5. Tspo Protects Mitochondrial Morphology and Function in Macrophages

**Figure 4:**
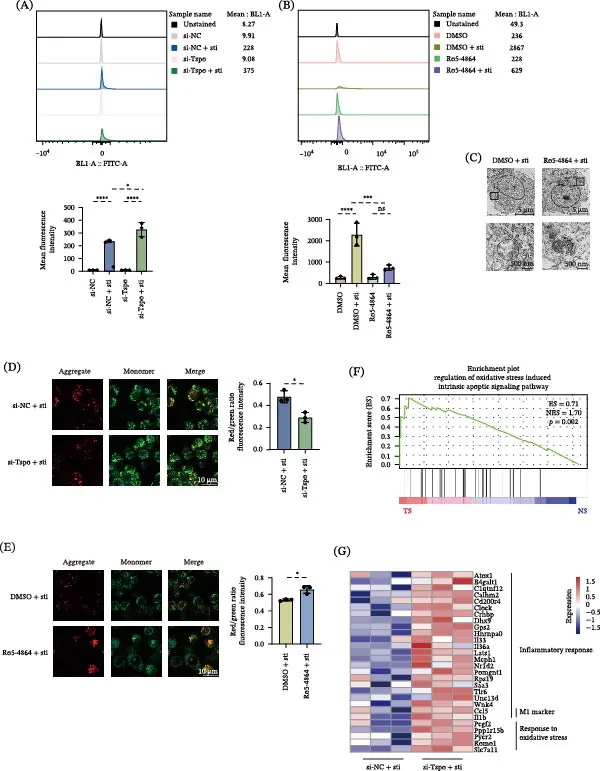
Relationship between Tspo and mitochondrial function and morphology. (A) RAW264.7 were transfected with si‐NC or si‐Tspo and treated with or without inflammatory stimulation. Flow cytometry was applied to detect DCFH‐DA fluorescence intensity, followed by quantification of MFI. (B) RAW264.7 were treated with DMSO or Ro5‐4864 and with or without inflammatory stimulation. Flow cytometry was applied to detect DCFH‐DA fluorescence intensity, followed by quantification of MFI. (C) Representative TEM images depicting the morphological alterations in RAW264.7 mitochondria following treatment with DMSO + sti and Ro5‐4864+sti. The lower sections of the images depict magnified regions corresponding to the upper images, which are enclosed within black boxes (upper section scale bar = 5 μm; lower section scale bar = 500 nm). (D–E) Detection of ΔΨm using JC‐1 probe. Red indicated JC‐1 aggregates, while green indicated JC‐1 monomer. The ΔΨm was determined by calculating the ration of red‐to‐green fluorescence intensity (scale bar = 10 μm). (F) GSEA analysis identifies enrichment scores for gene set of “Regulation of oxidative stress induced apoptotic signaling pathway”. (G) Heatmap displaying M1 markers, oxidative stress and inflammatory factors related genes upregulated in the si‐Tspo+sti group.  ^∗^ *p* value < 0.05;  ^∗∗∗^ *p* value < 0.001;  ^∗∗∗∗^ *p* value < 0.0001; ΔΨm, mitochondrial membrane potential; DEGs, differentially expressed genes; MFI, mean fluorescence intensity; ns, nonsignificant; si‐NC + sti/NS, transfected with siRNA negative control and subsequently stimulated with IFN‐γ and LPS; si‐Tspo+sti/TS, transfected with siRNA‐Tspo and subsequently stimulated with IFN‐γ and LPS; TEM, transmission electron microscope; unstained, the corresponding unstained control data.

As Tspo is a well‐characterized mitochondrial outer membrane protein [17], we further investigated its role in regulating mitochondrial function and morphology under inflammatory conditions. Given that mitochondria are the primary intracellular source of ROS, we first assessed ROS levels in macrophages using the DCFH‐DA fluorescent probe. Flow cytometry analysis showed that the downregulation of Tspo significantly increased ROS production in macrophages following inflammatory stimulation (Figure 4A); in contrast, pretreatment with the Tspo agonist Ro5‐4864 markedly reduced ROS levels under the same inflammatory conditions (Figure 4B).

We next evaluated the effect of Tspo on inflammation‐induced mitochondrial structural damage in RAW264.7 macrophages. Pretreatment with Ro5‐4864 exerted a protective effect: it attenuated inflammation‐induced mitochondrial membrane disruption, reduced cristae disorganization and mitochondrial swelling (Figure 4C), and maintained mitochondrial membrane potential (ΔΨ_m_) (Figure 4E). Conversely, Tspo downregulation exacerbated inflammatory injury to mitochondria, as evidenced by significant ΔΨ_m_ depolarization following stimulation (Figure 4D).

To further investigate the anti‐inflammatory effect of Tspo, we conducted RNA‐seq analysis of the si‐Tspo+stimulated (si‐Tspo+sti/TS) and si‐NC + stimulated (si‐NC + sti/NS) groups. GSEA was adopted to further analyze the RNA‐seq data. According to the GSEA result, it revealed that the downregulation of Tspo increased the enrichment score of “Regulation of oxidative stress induced apoptotic signaling pathway” (Figure 4F), which was correlated with the conclusion of our study that Tspo may play a protective response role during inflammation stimulation. Given that oxidative stress and inflammatory factors are the primary focus of this study, we displayed differentially expressed genes (DEGs) associated with “inflammatory response” and “response to oxidative stress” (Figure 4G). We found that Tspo knockdown amplified inflammatory and oxidative stress responses, accompanied by an upregulation of M1 macrophage markers (Figure 4G).

Collectively, these findings demonstrate that Tspo plays a critical role in preserving mitochondrial structural integrity and functional homeostasis in macrophages during inflammation.

### 2.6. Tspo as a Potential Therapeutic Target for Myocarditis

**Figure 5:**
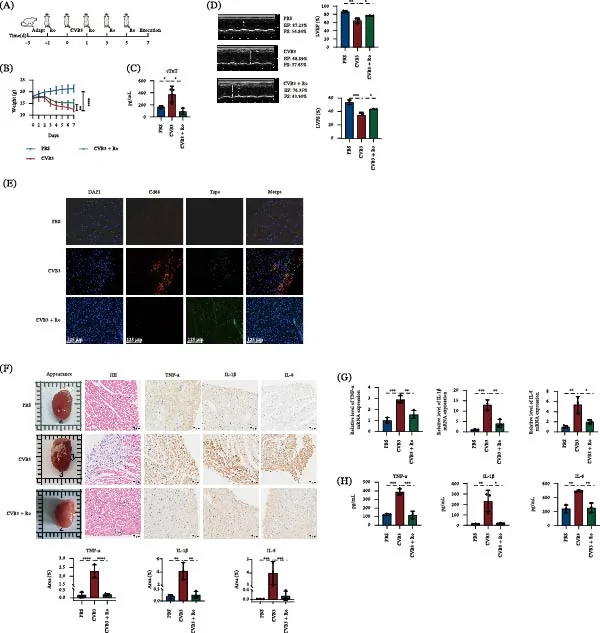
The administration of Ro5‐4864 in mice with CVB3‐induced myocarditis confers cardioprotection and attenuates the inflammatory response. (A) Schematic of the administration workflow of Ro5‐4864 in mice with CVB3‐induced myocarditis. (B) The daily weight changes of mice receiving different treatments were monitored for 8 days in three groups: PBS (*n* = 3), CVB3 (*n* = 3), and CVB3+Ro (*n* = 3). Intergroup difference was analyzed by *two-way ANOVA*. (C) The levels of cTnT in the serum of mice from three groups were quantified using ELISA (*n* = 3). (D) Representative short‐axis M‐mode echocardiography images of mice assigned to PBS, CVB3, and CVB3+Ro groups. The cardiac function was presented as %LVEF and %LVFS (*n* = 3). (E) Representative immunofluorescence images depicting that the administration of Ro5‐4864 could diminished the infiltration of macrophage (scale bar = 125 μm). (F) Representative images of heart tissues, including gross morphology, H&E‐stained sections, and IHC images. Positive staining area proportion (%Area) was calculated on IHC sections (scale bar = 50 μm; *n* = 3). (G) Relative mRNA expression of TNF‐α, IL‐1β and IL‐6 in heart tissues of mice from three groups (*n* = 3). (H) The levels of TNF‐α, IL‐1β and IL‐6 in the serum of mice from three groups were quantified using ELISA (*n* = 3).  ^∗^ *p* value < 0.05;  ^∗∗^ *p* value < 0.01;  ^∗∗∗^ *p* value < 0.001;  ^∗∗∗∗^ *p* value < 0.0001; CVB3+Ro, mice injected with CVB3 and Ro5‐4864; ELISA, enzyme‐linked immunosorbent assay; FS, fractional shortening; H&E, hematoxylin and eosin; IHC, immunohistochemistry; LVEF, left ventricular ejection fraction; Ro, Ro5‐4864.

To evaluate the potential of Tspo as a therapeutic target for myocarditis, we utilized a CVB3‐induced myocarditis mouse model. Mice were intraperitoneally administered Ro5‐4864, and the treatment protocol is outlined in Figure 5A. Treatment with the Tspo agonist significantly mitigated weight loss induced by CVB3 infection (Figure 5B). Additionally, CVB3 infection resulted in cardiac dysfunction, which was notably improved following Ro5‐4864 treatment. This improvement was evidenced by reduced plasma cTnT levels and enhanced cardiac function, as indicated by a significant increase in left ventricular ejection fraction (%LVEF) and left ventricular fractional shortening (%LVFS) (Figure 5B−D, *p* < 0.05). Histological analysis revealed a reduction in immune cell infiltration, as observed through heart morphology, H&E staining, and IF (Figure 5E,F). Correspondingly, Ro5‐4864 treatment decreased the expression of common proinflammatory cytokines, as shown by immunohistochemistry (IHC) and RT‐qPCR results (Figure 5F,G). Furthermore, enzyme‐linked immunosorbent assay (ELISA) analysis demonstrated reduced levels of inflammatory cytokines in the plasma of mice from the CVB3+Ro5‐4864 treatment group compared to the CVB3 group (Figure 5H, *p* < 0.05).

Collectively, these findings suggest that Ro5‐4864 treatment effectively alleviates CVB3‐induced myocarditis, supporting Tspo as a potential therapeutic target for myocarditis.

## 3. Discussion

Myocarditis, characterized by myocardial inflammation and tissue injury, is a major cause of heart failure in young individuals [13, 18], yet its pathogenesis remains incompletely understood [3, 5]. Current evidence suggests that VMC is driven by both direct viral injury and dysregulated host immune responses, with marked temporal and cellular heterogeneity throughout the disease progression [19]. In the early stage of infection, cardiomyocytes and cardiac‐resident immune cells recognize viral components through pattern‐recognition pathways, including Toll‐like receptors, RIG‐I‐like receptors, and the cGAS‐STING pathway, thereby inducing type I IFN and other inflammatory mediators to suppress viral replication and initiate antiviral defense [19–21]. This process is accompanied by the activation and recruitment of myeloid cells, dendritic cells, neutrophils, and stromal cells, which further amplify local inflammation.

In the present study, macrophage‐associated cellular and molecular alterations represented the most prominent immune features of this dataset, suggesting that myeloid remodeling may be a key pathological event at this stage. After analysis of the scRNA‐seq data, we also detected a modest expansion of T cells, indicating that adaptive immune activation participates in VMC, although to a limited extent. This is consistent with previous studies showing that T cells play context‐dependent roles in VMC: cytotoxic T cells contribute to viral clearance but may also exacerbate myocardial injury, whereas CD4^+^ T‐cell subsets, including Th1, Th17, and regulatory T cells, exert distinct proinflammatory or immunomodulatory effects depending on disease stage and the local microenvironment [19, 22, 23]. The relatively small magnitude of T‐cell expansion observed here is more likely related to the sampling characteristics of GSE174458 than to a minor role of T cells in pathogenesis. Because samples were collected on day 21 after CVB3 infection, corresponding to a postinfectious stage, this dataset likely reflects the transition from viral clearance to persistent inflammation and tissue remodeling rather than the peak of acute T‐cell infiltration [24]. In addition, because VMC lesions are often focal, locally enriched T‐cell signals may have been diluted in whole‐heart dissociation‐based scRNA‐seq analysis, as supported by spatial transcriptomic evidence showing recruitment of cytotoxic T cells to lesion areas by inflamed endothelial cells [25]. Taken together, these findings support the involvement of T cells in disease progression, while indicating that the overall transcriptomic landscape at this stage remains predominantly characterized by macrophage‐associated alterations.

Mitochondrial dysfunction has long been recognized as a hallmark of myocarditis progression, primarily through excessive ROS generation and impaired energy metabolism. Previous studies showed that CVB3 infection disrupts mitochondrial function and promotes oxidative stress [26–28]. Mitochondrial injury can promote the release of mitochondrial DNA, thereby activating the cGAS‐STING‐TBK1‐IRF3 axis and enhancing type I IFN expression [29, 30]. Therefore, type I IFN signaling is recognized as a central antiviral pathway in myocarditis and may provide an important mechanistic link between immune activation and mitochondrial stress [20, 21, 31]. Consistent with this, our gene set variation analysis (GSVA) analysis of the GSE248521 dataset showed significant upregulation of the type I IFN response pathway in the myocarditis group (Figure S9), and two of the five hub genes identified in this study, Cmpk2 and Ifi27, are interferon‐responsive genes. However, their direct roles in myocarditis remain unclear.

Through integrative bioinformatics analysis and experimental validation, we further focused on the mitochondria‐associated protein Tspo. Tspo has been implicated in multiple inflammatory conditions, including neuroinflammation, atherosclerosis, and myocardial inflammation [32–37]. Besides, it has been demonstrated that Tspo deficiency aggravates GSDMD‐mediated macrophage pyroptosis and exacerbates DSS‐induced colitis [38]. Moreover, Kikutani et al. [39] reported that Tspo gene deletion amplifies inflammatory injury in postsepsis syndrome.

Mechanistically, Tspo is localized to the OMM [17], where it contributes to the maintenance of mitochondrial membrane potential and the prevention of ROS overproduction. Transmission electron microscope (TEM) and JC‐1 analyses confirmed that Tspo preserves mitochondrial morphology and potential under inflammatory conditions, whereas Tspo deficiency led to mitochondrial fragmentation‐a phenotype consistent with previous findings that restoring Tspo rescues mitochondrial architecture [38, 40]. Given that mitochondrial fragmentation promotes ROS accumulation and loss of membrane potential [41–43], our results support a model in which Tspo maintains mitochondrial integrity to suppress downstream oxidative and inflammatory cascades. At the immune‐cell level, reduced Tspo expression in macrophages promotes polarization toward the proinflammatory M1 phenotype [38], further amplifying tissue inflammation. Consistent with this, treatment with the Tspo agonist Ro5‐4864 inhibited ROS production and cytokine release from macrophages [44, 45]. Furthermore, our study revealed that Tspo functions as a protective response factor, and administration of its agonist, Ro5‐4864, exhibits therapeutic effects in a mouse model of myocarditis. Together, these findings delineate a Tspo‐mitochondria‐ROS/inflammation axis as a key determinant of myocardial injury severity.

In conclusion, our findings reveal that Tspo in macrophages as a pivotal mitochondria‐associated protective response factor that preserves mitochondrial morphology, limits oxidative stress, and restrains inflammation during myocarditis. By integrating bioinformatics, molecular, and functional analyses, this study reveals that the Tspo‐mitochondria axis regulates inflammatory injury in the heart. Besides, this study is the first to demonstrate that the Tspo agonist Ro5‐4864 exerts a therapeutic effect in CVB3‐induced mouse model of myocarditis. Given the lack of targeted therapies for myocarditis, pharmacological activation of Tspo represents a promising avenue for future therapeutic development.

Several limitations of this study should be acknowledged. First, the presence of erythrocytes in the scRNA‐seq samples made it difficult to distinguish circulating immune cells from tissue‐resident cardiac cells, which may have affected the interpretation of cellular composition, especially for nonimmune populations. Moreover, because the scRNA‐seq dataset included only two biological replicates per group, sample‐level comparisons should be interpreted cautiously and regarded mainly as supportive evidence for cell‐type enrichment. Second, although our in vitro data supported a protective role for Tspo, more physiologically relevant models, including cardiomyocyte–macrophage coculture systems and direct viral infection models, are needed to further validate these findings. Third, our study relied primarily on pharmacological modulation of Tspo, without genetic loss‐of‐function approaches such as myeloid‐specific conditional knockout mice, thereby limiting causal in vivo validation. Fourth, while our analyses implicated type I IFN signaling as a potential link between immune activation and mitochondrial stress, this pathway was not systematically validated in cardiac tissues or in vitro models. Finally, the precise molecular mechanisms by which Tspo regulates mitochondrial homeostasis and inflammatory signaling, as well as the clinical relevance of these findings in human myocarditis, remain to be determined.

## 4. Material and Methods

### 4.1. Data Acquisition

Expression data of myocarditis were selected from the GEO (http://www.ncbi.nlm.nih.gov/geo/) database, which are all publicly available. GSE248521 [46], GSE35182 [35], and GSE53607 [47] contain expression data from heart tissues. The training set (GSE248521), generated using the GPL19057 Illumina NextSeq 500 platform (*Mus musculus*), comprises samples from male C57BL/6;129P2 mice (7‐ to 9‐week‐old) infected intraperitoneally with 5 × 10^5^ plaque‐forming units (PFU) of CVB3 (Nancy strain). From this dataset, 6 acute myocarditis models (6 days postinfection [dpi]) and 6 matched controls were selected for analysis. The validation sets included two independent datasets, both generated using the GPL6246 Affymetrix Mouse Gene 1.0 ST Array platform (transcript [gene] version): GSE35182 contains samples from BALB/c mice (male and female, 6‐ to 8‐week‐old) infected with 10^3^ PFU of heart‐passaged CVB3 (diluted in sterile PBS), including 6 acute myocarditis (10 dpi) and 6 matched control; GSE53607 includes samples from male C3H/HeNTac mice (5‐week‐old) infected with 2 × 10^7^ PFUs of the DA strain of TMEV intraperitoneally, consisting of 5 acute myocarditis (7 dpi) and 5 matched control.

In addition, GSE174458 [24] encompasses scRNA‐seq data derived from heart tissues of CVB3‐infected male A/J mice (6‐ to 8‐week‐old and 9‐ to 11‐week‐old) at 21 dpi and was generated using the GPL21103 Illumina HiSeq 4000 platform. Mice were intraperitoneally inoculated with 10^4^ 50% tissue culture infectious dose (TCID_50_) of CVB3 Nancy strain (ATCC VR‐30) in 200 μL PBS, along with age‐matched uninfected controls (2 biological replicate pairs per condition). Detailed information on these datasets is displayed in Table 2.

**Table 2:** Detailed information of the four datasets.

Dataset ID	Tissue	No. of samples	GPL ID	RNA‐seq	Usage here	References
GSE248521	Heart	6myocarditis, 6control	GPL19057	mRNA‐seq	Training set	Stoffers et al. [46]
GSE35182	Heart	6myocarditis, 6control	GPL6246	mRNA‐seq	Validation set	Coronado et al. [35]
GSE53607	Heart	5myocarditis, 5control	GPL6246	mRNA‐seq	Validation set	Omura et al. [47]
GSE174458	Heart	2myocarditis, 2control	GPL21103	scRNA‐seq	Validation set	Lasrado et al. [24]

In addition, a total of 1140 mouse mitochondrial metabolism‐related genes were obtained from the MitoCarta3.0 database (http://www.broadinstitute.org/mitocarta) [48]. To retrieve a comprehensive set of human myocarditis‐associated genes, GeneCards (https://www.genecards.org/) [49] was employed by querying the term “myocarditis”, resulting in the acquisition of 882 protein‐coding genes (Table S1).

### 4.2. Construction of Weighted Gene Coexpression Network

The expression data of all genes was uploaded to construct the weighted gene coexpression network using “WGCNA” (v.1.73) package [50]. WGCNA can be used to identify highly correlated gene clusters, summarize these clusters using module eigengenes or intramodular hub genes, associate these modules with external sample traits, and calculate MM [50]. The soft thresholding power β was determined using the pickSoftThreshold function, while the hclust function was utilized to identify gene module. Gene modules were defined as groups of genes exhibiting similar expression patterns, with minModuleSize set as 200. The associations between modules and disease status were assessed through *Pearson* correlation coefficients, and modules displaying the most positive correlation coefficients were retained for further analysis, which were deemed as hub module (gray module was excluded). Finally, GS and MM were computed to identify key genes within modules based on a cut‐off criterion of |GS| ≥ 0.50 and |MM| ≥ 0.60 (Table S4), which were defined as mouse‐derived myocarditis‐associated genes.

### 4.3. Enrichment Analysis

To explore the type I IFN response in myocarditis, GSVA [43] was performed using the “HALLMARK_INTERFERON_ALPHA_RESPONSE” gene set from the “mh.all.v2026.1.Mm.symbols” (https://www.gsea-msigdb.org/ gsea/msigdb/mouse/geneset/HALLMARKINTERFERON_ALPHA_RESPONSE).

To understand the function of key genes in hub module, we conduct functional enrichment analysis employing Metascape (https://metascape.org/gp/index.html#/main/step1) [51], which includes biological processes in Gene Ontology (GO) and pathways in KEGG. *p* values  < 0.05 were considered statistically significant. Top 10 terms sorted by ‐log_10_ (*p* value) were selected to display in the form of bar plot.

### 4.4. The Selection and Validation of Hub Genes

DEGs between myocarditis and control in the training set were defined using a significance threshold of *p* value  < 0.05 and |log_2_FC| ≥ 0.5, which was calculated through “limma” (v.3.62) R package. To identify mouse‐derived MitoMYCGs, we intersected differentially expressed mouse‐derived myocarditis‐associated genes obtained from WGCNA with MtGs from the MitoCarta3.0 database.

To further evaluate the relative contribution of 11 candidate genes to the discrimination between groups, RF [52] and SHAP [53] analyses were performed based on the same predefined candidate gene set. The classification task was a binary comparison between myocarditis and control samples. The expression matrix and phenotype annotation were matched by sample ID, and the data were reshaped into a sample‐by‐gene matrix. Phenotype was used as the class label, with 0 indicating control and 1 indicating myocarditis. A RF model was built in R using the 11 candidate genes as features, and variable importance was used to rank their contributions. For model interpretability, SHAP analysis was performed in Python on the same 11 genes using a “RandomForestClassifier”. SHAP values for the myocarditis class were calculated with “TreeExplainer”, and the mean absolute SHAP values were used for ranking and visualization.

Given the limited sample size, these analyses were exploratory. No independent test set or external validation cohort was available. To reduce model complexity and avoid overinterpretation, only 11 candidate genes were used as input features. Random seeds were fixed in both R and Python to improve reproducibility. Therefore, the RF and SHAP results were interpreted as internal exploratory evidence rather than proof of robust predictive performance or external generalizability.

Subsequently, the remaining genes were validated in GSE35182 and GSE53607 using “limma” analysis, considering *p* values  < 0.05 as statistically significant.

### 4.5. Cluster Analysis of scRNA‐Seq

“Seurat” (v4.2.3) R package was used to process scRNA‐seq data from two pairs of myocarditis heart tissues in GSE174458. After quality control (200 < nFeature_RNA < 3000, percent.mt < 10%) and doublet removal using “scDblFinder” [54], the four samples were merged and normalized. The top 2000 highly variable genes were used for dimensionality reduction, clustering, and UMAP visualization based on the top 30 principal components. Cell types were manually annotated according to canonical marker genes. Differential expression of hub genes between myocarditis and control groups was assessed across all cells and within the macrophage cluster using “FindMarkers” with the Wilcoxon rank‐sum test. Genes with *p* < 0.05 were considered significant, and detailed results are shown in Table 1.

### 4.6. Cell Culture, Transfection, and Administration of Agonist

RAW264.7, a mouse macrophage line (purchased from HyCyte Technology), was cultured in DMEM (Gibco, US) with 10% fetal bovine serum (FBS, Gibco, US) and 1% penicillin/streptomycin (Invitrogen, California, USA), 1% glutamate (HyCyte Technology, Suzhou, China), and 1% sodium pyruvate (HyCyte Technology, Suzhou, China) at 37°C in 5% CO_2_. The cells were seeded at a density of 1 × 10^6^ cells/well in six‐well plates and incubated for ~24 h until reaching a confluency of 60%‐80%. Subsequently, the cells were treated with LPS (Sigma‐Aldrich, St. Louis, Michigan, USA; #L2880; 1 μg/mL) and IFN‐γ (PeproTech, New Jersey, USA; #315‐05; 20 ng/mL) for another 24 h. The mRNA expression of proinflammatory cytokines, including IL‐1β, IL‐6, and TNF‐α was detected for validation.

The downregulation experiment was accomplished by transfecting RAW264.7 cells with si‐Tspo and si‐NC (Genomeditech Co., Ltd., China), respectively, at a concentration of 100 nM using GP‐Transfect Mate RNAiMAX reagent (GenePharma, Shanghai, China; #G04008) in accordance with the manufacturer’s instructions. The Tspo function was further investigated by incubating cells with its agonist, 4^′^‐chlorodiazepam (Ro‐54864; MedChemExpress, New Jersey, USA; #HY‐124268), at a concentration of 1 mM for 1 h at 37°C in 5% CO_2_, followed by the addition of an inflammatory stimulation.

### 4.7. Animal Model and Treatment

Male BALB/c mice (4‐week‐old) were obtained from Beijing Vital River Laboratory Animal Technology Co., Ltd. and housed under specific pathogen‐free conditions (22‐25°C, 50%‐60% humidity, and 12 h light/dark cycle) with free access to food and water. After 3 days of acclimatization, mice were randomly assigned to the control, CVB3, or CVB3+Ro5‐4864 group (*n* = 3/group). All animals were included in the final analysis. This was an exploratory animal study, and sample size was determined based on prior experience and the principle of reduction. All procedures were approved by the Animal Ethics Committee of Shandong University and Shandong Provincial Hospital (Number SDU‐IACUC‐25046) and conducted in accordance with the ARRIVE 2.0 guidelines [55] and the 3R principles. Mice were monitored daily, and no deaths or humane endpoints occurred before the planned endpoint.

CVB3 (Nancy strain) was propagated in Hep‐2 cells, and viral titers were determined by TCID_50_ assay. Mice in the CVB3 and CVB3+Ro5‐4864 groups received an intraperitoneal injection of 0.2 mL PBS containing 1 × 10^5^ TCID_50_ CVB3, whereas control mice received an equal volume of PBS. Ro5‐4864 was dissolved in vehicle (10% DMSO, 40% PEG300, 5% Tween‐80, and 45% normal saline) and administered intraperitoneally at 1 mg/kg 24 h before CVB3 infection and every 2 days thereafter.

All invasive procedures were performed under isoflurane anesthesia (induction: 2%‐3% and maintenance: 1.5%‐2.0%). On day 7 postinfection, transthoracic echocardiography was performed using an M‐mode ultrasound imaging system (SiliconWave 60, Suzhou Color Medical, China) to assess left ventricular systolic function. Body temperature was maintained with a heating pad, and images were analyzed in a blinded manner. Under deep anesthesia and loss of pain reflex, terminal blood was collected by cardiac puncture, followed by euthanasia with isoflurane overdose. After thoracotomy and perfusion with sterile PBS, hearts were harvested, fixed in 4% paraformaldehyde, paraffin‐embedded, sectioned at 5 μm, and subjected to hematoxylin and eosin staining for histopathological analysis [56]. Blood was collected into EDTA‐Na_2_ tubes, and plasma was isolated for measurement of inflammatory cytokines and myocardial injury markers.

### 4.8. Immunofluorescence and IHC

After dewaxing and rehydration, paraffin sections were subjected to antigen retrieval by microwave heating, then incubated with 3% hydrogen peroxide to block the endogenous peroxidase, and subsequently sealed with 3% bull serum albumin. For immunofluorescence analysis, sections were labeled with primary antibodies against Cd68 (Servicebio technology Co.,Ltd., Wuhan, China; #GB113109; 1:1000) and Tspo (Abcam, Cambridge, UN; #ab109497; 1:1000) and then incubated with secondary antibodies coupled to horseradish peroxidase (HRP; Servicebio; #GB23303; 1:500) with additional staining using CY3‐Tyramide (Servicebio; #G1223; 1:500) and Alexa Fluor 488 (Servicebio; #GB25303; 1:400) at room temperature for 50 min, respectively. Nuclei stained with 4^′^,6‐diamidino‐2‐phenylindole (DAPI; Sigma‐Aldrich; #D9542), and the samples were observed by EVOS M7000 fluorescence microscope (Invitrogen, Massachusetts, USA). For IHC analysis, sections were labeled with primary antibodies against IL‐1β (Servicebio; #GB11188; 1:800), IL‐6 (Servicebio; #GB11188; 1:200), and TNF‐α (Servicebio; #GB11188; 1:200). The sections were subsequently incubated with HRP‐coupled secondary antibodies comprising goat antirabbit IgG (Servicebio; #GB23303; 1:200) at room temperature for 50 min. Following this, the sections were stained using DAB, and the nuclei were restained with hematoxylin. Finally, the samples were visualized under light microscopy.

### 4.9. ELISA of Plasma Samples

The plasma samples were centrifuged at 4°C, 1000 g for 15 min within 30 min after collection, and the supernatant was gathered for detection of inflammatory cytokines (IL‐1β, IL‐6, and TNF‐α) and cardiac biomarker cardiac troponin (cTnT). Mouse ELISA kit (Elabscience, Houston, Texas, USA) was used for the detection of IL‐1β (#E‐EL‐M0037), IL‐6 (#E‐EL‐M0044), TNF‐α (#E‐EL‐M3063), and cTnT (#E‐EL‐M1801) according to the manufacturer’s instructions.

### 4.10. In Vivo Flow Cytometry

Mouse cardiac tissues were isolated and dissociated into single‐cell suspensions. Cells were stained with APC‐conjugated anti‐CD45 (Biolegend, San Diego, USA; #103111) and PE‐conjugated anti‐F4/80 (Biolegend; #123109) and analyzed by flow cytometry (Cytek Aurora, Cytek Biosciences, Fremont, California, USA). Data were processed using FlowJo 10.8.1 software. The positivity gates for both Cd45 and F4/80 were set at 10^3^, as shown in Figure S6. Immune cells were identified as Cd45^+^ populations, and the proportion of F4/80^+^ macrophages was subsequently quantified within this gate.

### 4.11. RT‐qPCR

Total RNA was extracted from cultured cells and heart tissues with TRIzol (Vazyme Biotech Co., Ltd., Nanjing, China) in accordance with the manufacturer’s instructions and was reversely transcribed into cDNA with a reverse transcription kit (Vazyme Biotech Co., Ltd.). Then, cDNA was amplified with specific real‐time PCR primers using SYBR green (Vazyme Biotech Co., Ltd.). The levels of mRNA were analyzed using the 2^−ΔΔCt^ method [57] and normalized against Actin. RT‐qPCR was performed for verification. All the sequence are displayed in Table S2.

### 4.12. Western Blot

Cells were lysed in RIPA lysis buffer (Beyotime, Shanghai, China) with the addition of 1 mM PMSF (Beyotime, Shanghai, China) on ice. After centrifugation at 4°C, 12,000 RPM for 5 min, the protein content in the supernatant was quantified using the BCA assay kit (ACE Biotechnology, Nanjing, China). The protein samples were then added into the 12% polyacrylamide gels for electrophoresis and subsequently transferred onto a polyvinylidene fluoride membrane (Merck Millipore, Bedford, MA, USA). The membrane was blocked with 5% skim milk dissolved in TBST for 1 h before being incubated overnight at 4°C with respective primary antibodies including anti‐Tspo (1:5000) and anti‐β‐tubulin (CST Biological Technology Co., Ltd., Boston, USA; #2128; 1:1000). HRP‐conjugated goat antirabbit IgG (Jingjie PTM BioLab Co., Inc., Hangzhou, China; #PTM‐6261; 1:2000) was then applied to the membranes for another hour at room temperature. Relative protein expression levels were determined using ImageJ software (National Institutes of Health, Bethesda, MD, USA).

### 4.13. RNA Sequencing (RNA‐Seq)

RNA samples extracted from si‐NC+sti/NS and si‐Tspo+sti/TS in the knockdown experiment were submitted to Berry Genomics (Beijing, China) for transcriptomic sequencing to further explore the mechanism of Tspo in macrophages. A total of 56,980 mRNAs were detected using Illumina NovaSeq 6000 high‐throughput sequencing platform. The difference between si‐NC + sti group and si‐Tspo+sti group was analyzed by edgeR. Genes with counts ≥10 in at least one sample were retained, and the data were converted to FPKM, resulting in 14,469 genes. GSEA analysis was then performed using the gene set “m5.go.bp.v2025.1.Mm.symbols”. Gene sets related to inflammation and oxidative stress were derived from “m5.go.bp.v2025.1.Mm.symbols” (https://www.gsea-msigdb.org/gsea/msigdb/mouse/geneset/GOBP_INFLAMMATORY_RESPONSE; https://www.gsea-msigdb.org/gsea/msigdb /mouse/geneset/GOBP_RESPONSE_TO_OXIDATIVE_STRESS). M1 markers were obtained from previous study [58]. Among these, genes upregulated in si‐Tspo+sti group were visualized in the form of heatmap.

### 4.14. Measurement of Cellular ROS and Membrane Potential (ΔΨm)

RAW264.7 cells with different treatments were stained with dichlorofluorescein diacetate (DCFH‐DA) green dye (Beyotime Biotechnology, China; #S0033S; 20 μM) for 45 min at 37°C. After carefully washing and resuspension in PBS, the fluorescent intensity of cells was detected with flow cytometry (AttuneNxT, Thermo Fisher Scientific, Massachusetts, USA), with data analyzed using FlowJo software. The mean fluorescence intensity (MFI) of ROS staining was measured to quantitatively assess intracellular ROS levels.

The changes in mitochondrial ΔΨm of RAW264.7 under different conditions were assessed by JC‐1 (MedChemExpress; #HY‐15534; 1 μg/mL) fluorescence probe at 37°C for 20 min. In normal physiological conditions, JC‐1 undergoes aggregation to form a polymer and emits red fluorescence; however, under low ΔΨm, JC‐1 exists as monomers and exhibits green fluorescence. Consequently, the ratio of red to green fluorescence intensity serves as an indicator for assessing ΔΨm. Prior to image, the cells were subjected to a triple wash with PBS. Fluorescent images were acquired using Cell Discovery 7 and analyzed accordingly. Additionally, flow cytometry was utilized to measure fluorescent intensity of cells, and FlowJo software was used to analyze the obtained data. All assays were performed in accordance with the protocol provided by the manufacturer.

### 4.15. TEM

RAW264.7 cells in DMSO+sti and Ro5‐4864+sti groups were fixed in 2.5% glutaraldehyde at 37°C for 30 min, which were then sent to Wuhan Servicebio technology Co., Ltd. for TEM analysis. The mitochondrial ultrastructural structure was observed under TEM.

### 4.16. Statistical Analyses

For validation in datasets and experiments, the differences in genes between groups were presented as mean ± standard deviation (SD), and analyzed by *unpaired Student’s t test* (two groups, the differences in GSE174458 were analyzed by *Mann–Whitney U test*) or *one/two-way ANOVA* (for more than two groups) using GraphPad Prism v.10.1.2, and were considered statistically significant if *p* values  < 0.05. Electronic laboratory notebook was not used.

**Nomenclature**
AUC: Area under the curve
BPs: Biological processes
CVB3: Coxsackievirus B3
CVB3+Ro: Mice injected with CVB3 and Ro5‐4864
DAPI: 4^′^,6‐diamidino‐2‐phenylindole
DCFH‐DA: Dichlorofluorescein diacetate
DEGs: Differentially expressed genes
DMSO+sti: Treatment with DMSO and subsequently stimulated with IFN‐γ and LPS
dpi: Days post infection
Echo: Echocardiograms
ELISA: Enzyme‐linked immunosorbent assay
FC: Fold change
GEO: Gene expression omnibus
GO: Gene Ontology
GS: Gene significance
GSEA: Gene set enrichment analysis
GSVA: Gene set variation analysis
H&E: Haemtoxylin and Eosin
HRP: Horseradish peroxidase
IHC: Immunohistochemistry
IFN: Interferon
IL: Interleukin
KEGG: Kyoto Encyclopedia of Genes and Genomes
LPS: Lipopolysaccharide
LVEF: Left ventricular ejection fraction
LVFS: Left ventricular fractional shortening
MFI: Mean fluorescence intensity
MitoMYCGs: Mitochondrial metabolism‐related genes related to myocarditis
MM: Module memberships
MtGs: Mitochondrial metabolism‐related genes
NC: Negative control
nCount_RNA: The sum of all gene expression measured in each cell
nFeature_RNA: The number of genes per cell
OXPHOS: Oxidative phosphorylation
percent.mt: The percentage of mitochondrial gene
RT‐qPCR/qPCR: Reverse transcription quantitative PCR
RF: Random forest
RNA‐seq: RNA sequencing
ROC: Receiver operating characteristic
ROS: Reactive oxygen species
Ro5‐4864+sti: Treatment with Ro5−4864 and subsequently stimulated with IFN‐γ and LPS;
scRNA‐seq: Single‐cell RNA‐sequencing
SD: Standard deviation
SHAP: SHapley Additive exPlanations
siRNA: Small interfering RNA
si‐NC: Negative control
si‐NC+sti: Transfected with siRNA negative control and subsequently stimulated with IFN‐γ and LPS
si‐Tspo: siRNA targeting Tspo
si‐Tspo+sti: Transfected with siRNA‐Tspo and subsequently stimulated with IFN‐γ and LPS
TCID_50_: 50% tissue culture infectious dose
TEM: Transmission electron microscope
TNF: Tumor necrosis factor
Tspo: Translocator protein 18‐kDa
VMC: Viral myocarditis
WGCNA: Weighted gene coexpression network analysis
ΔΨm: Mitochondrial membrane potential.

## Author Contributions

**Yaxue Xie and Yingnan You**: conceptualization, data curation, formal analysis, investigation, methodology, project administration, resources, software, writing – original draft, writing – review and editing. **Yanjie Jiang and Xiuyun Duan**: supervision, validation, writing – review and editing. **Bo Han**: funding acquisition, project administration, writing – review and editing.

## Funding

This study was supported by the Special Expert of Taishan Scholars (Grant 201511099), the Jinan Science and Technology Development Plan (Grant 202134015), Natural Science Foundation of Shandong Province (Grant ZR2023MH052), and Shandong Provincial Natural Science Foundation for Youths (Grant ZR2023QH310).

## Disclosure

The AI tool (ChatGPT) was not used for data collection, experimental design, or data analysis. All AI‐generated content was carefully reviewed, verified against the original data, and revised by the authors in light of relevant literature and the study findings. The authors take full responsibility for the accuracy, integrity, and scientific content of the final manuscript. All authors approved the final version of the manuscript.

## Ethics Statement

All animal experiments followed the guidelines outlined in the guide for the laboratory animals and were approved by both the Animal Ethics Committee of Shandong University and Shandong Provincial Hospital (SDU‐IACUC‐25046).

## Conflicts of Interest

The authors declare no conflicts of interest.

## Supporting Information

Additional supporting information can be found online in the Supporting Information section.

## Supporting information


**Supporting Information** Figure S1: Weighted gene coexpression network analysis. The cluster dendrogram depicts the distribution of genes with their corresponding module colors, encompassing a total of 16 modules. Soft threshold analysis is employed to determine the scale‐free fit index of network topology. Figure S2: Quality control of GSE174458. nCount_RNA: the sum of all gene expression measured in each cell; nFeature_RNA: The number of genes per cell; percent.mt: the percentage of mitochondrial gene. Figure S3: The expression and distribution of canonical cell markers are exhibited on the bubble plot. Figure S4: Sample‐level analysis of Tspo expression across cell types. Mean Tspo expression was calculated for each cell type in each biological sample. (Vascular‐like mixed population was not retained for C2 after quality control and cell‐type annotation). C1/2: sample 1/2 in control group; M1/2: sample 1/2 in myocarditis group. Figure S5: RT‐qPCR of hub genes in the heart tissue of CVB3‐induced myocarditis.  ^∗∗∗^ *p* value < 0.001,  ^∗∗∗∗^ *p*value < 0.0001. Figure S6: Gating strategy for flow cytometric analysis. Representative flow cytometry gating strategy used to identify cell populations of interest. Briefly, singlets were first gated from cells after exclusion of debris and doublets. The positivity gates for both Cd45 and F4/80 were defined at 10³ according to the unstained control. Figure S7: RT‐qPCR of proinflammatory cytokines in RAW264.7 cells.  ^∗∗^ *p* value < 0.01,  ^∗∗∗^ *p* value < 0.001. Figure S8: Efficiency of transfection. Compared with macrophages transfected with si‐NC, the expression of Tspo in those transfected with si‐Tspo was significantly decreased at both the mRNA (A) and protein (B) levels (*n* = 3).  ^∗∗^ *p* value < 0.01; si‐NC: siRNA negative control; si‐Tspo: siRNA targeting Tspo. Figure S9: GSVA score of type I IFN response between myocarditis and control.  ^∗∗^ *p* value < 0.01. Table S1: Human myocarditis related genes from Genecards. Table S2: RNA sequences for RT‐qPCR and transfection. Table S3: The percentage of 8 cell types in the myocarditis and control groups. myocarditis‐control: the difference value in cell proportion between myocarditis and control groups. Table S4: Detailed WGCNA results of genes within the yellow module.

## Data Availability

The original microarray data are available at Gene Expression Omnibus (https://www.ncbi.nlm.nih.gov/geo/). All data obtained in this study are contained in the figures and tables (Figures 1‐5; Tables 1 and 2; Figures S1‐S9; Tables S1–S4). The RNA‐seq data of si‐NC+sti and si‐Tspo+sti have been uploaded to GSE275137. Supporting Information is provided in the supporting file.
